# Interplay between type IV pili activity and exopolysaccharides secretion controls motility patterns in single cells of *Myxococcus xanthus*

**DOI:** 10.1038/srep17790

**Published:** 2016-01-29

**Authors:** Wei Hu, Maxsim L. Gibiansky, Jing Wang, Chuandong Wang, Renate Lux, Yuezhong Li, Gerard C. L. Wong, Wenyuan Shi

**Affiliations:** 1 State Key Laboratory of Microbial Technology, School of Life Science, Shandong University, Jinan, Shandong 250100, China; 2 School of Dentistry, University of California, Los Angeles, CA 90095, USA; 3 Department of Bioengineering, University of California, Los Angeles, CA 90095, USA

## Abstract

*Myxococcus xanthus* performs coordinated social motility of cell groups through the extension and retraction of type IV pili (TFP) on solid surfaces, which requires both TFP and exopolysaccharides (EPS). By submerging cells in a liquid medium containing 1% methylcellulose, *M. xanthus* TFP-driven motility was induced in isolated cells and independently of EPS. We measured and analyzed the movements of cells using community tracking algorithms, which combine single-cell resolution with statistics from large sample populations. Cells without significant multi-cellular social interactions have surprisingly complex behaviors: EPS^−^ cells exhibited a pronounced increase in the tendency to stand vertically and moved with qualitatively different characteristics than other cells. A decrease in the EPS secretion of cells correlates with a higher instantaneous velocity, but with lower directional persistence in trajectories. Moreover, EPS^−^ cells do not adhere to the surface as strongly as wild-type and EPS overproducing cells, and display a greater tendency to have large deviations between the direction of movement and the cell axis, with cell velocity showing only minimal dependence on the direction of movement. The emerging picture is that EPS does not simply provide rheological resistance to a single mechanism but rather that the availability of EPS impacts motility pattern.

Cellular motility provides bacteria with the capacity to actively seek out favorable environments and avoid hazardous situations, thereby facilitating growth and survival in natural habitats^1^. Some bacterial species have evolved motility mechanisms that allow cells to move along the direction of their long axis on solid surfaces without the aid of flagella^2^. In *Myxococcus xanthus*, a Gram-negative soil bacterium, flagella-independent motility is regulated by two separate adventurous (A) and social (S) systems^3^,^4^. S motility was initially described as the coordinated movement of large cell groups as discovered through the observations of the cells on agar surfaces^3^, and the force-generating mechanism involved in S motility has been shown to be the extension and retraction of type IV pili (TFP)^5^,^6^, which is mechanistically equivalent to the twitching motility in *Pseudomonas aeruginosa* and *Neisseria gonorrhoeae*^7^. It has been further demonstrated that the retraction of TFP is dependent on the interaction of TFP with exopolysaccharides (EPS)^8^, which is the reason S motility requires cell groups for movement and isolated A^−^S^+^ single-cells are not motile on agar surfaces. S motility is inherently a multi-cell phenomenon, so it is not clear how EPS impacts single-cell motility, or how single-cell motility is modified when more cells are involved.

It is known that *M. xanthus* S-motile (A^−^S^+^) cells are able to move as isolated cells on polystyrene surfaces when they are submerged in a highly viscous medium containing 1% methylcellulose^6^. Interestingly, the mutants defective in EPS production are found to perform TFP-dependent motility in this system^9^, although EPS is absolutely required for *M. xanthus* S motility on agar^10^,^11^. It has been proposed that the interactions between TFP and polystyrene surfaces are favored by methylcellulose, which may eliminate the requirement for EPS and enable TFP-dependent single-cell motility^9^. Previous studies manually tracked a small number of isolated cells in 1% methylcellulose. EPS^−^ cells, *e.g.*, SW504 (Δ*difA*) cells, exhibited a faster motion than EPS^+^ cells, *e.g.*, wild-type DK1622 cells^9^, which suggests that EPS may also affect the single-cell S motility in *M. xanthus*. Recent work has shown that the relation between EPS and TFP motility can be complex in *P. aeruginosa*^12^,^13^,^14^,^15^; therefore, it is likely that EPS may influence single-cell motility parameters other than cell speed. However, it is not clear whether *M. xanthus* can combine TFP activity with EPS production to produce different motility outcomes. Although tracking of single cells could be illuminating, the ability to track large populations should be useful for the time-resolved analysis of the underlying biological mechanisms of cell motility^16^.

In this study, we leverage recent advances in the tracking of early *P. aeruginosa* biofilm communities to combine single-cell resolution with large sample populations in the motility analysis of *M. xanthus*^12^. Using community tracking algorithms^13^,^17^,^18^, the motility histories of individual *M. xanthus* cells can be extracted by translating video microscopy movies into searchable databases of cell behavior, and motility patterns can be identified by tracking every cell in the database. Thus, we quantitatively characterized TFP-mediated single-cell motility of *M. xanthus* and correlated the differences in motility pattern to EPS production.

## Results

### Horizontal *M. xanthus* cells with different amount of secreted EPS exhibit different characteristics in single-cell S motility

**Figure 1:**
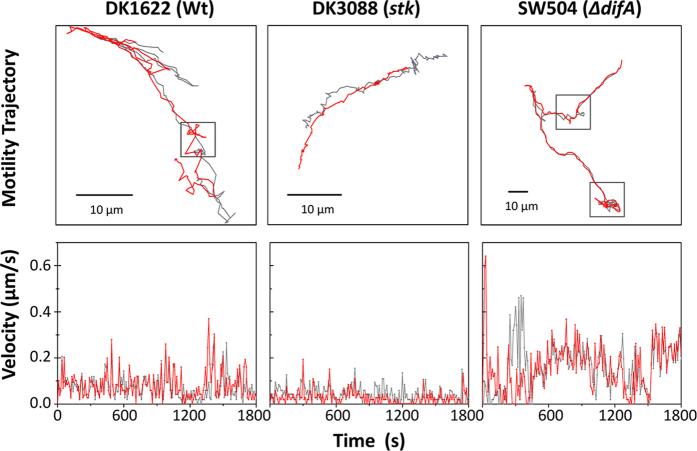
Representative trajectory and velocity profile for an isolated *M. xanthus* cell in 1% methylcellulose. DK1622 (Wt, EPS^+^), DK3088 (*stk*, EPS^++^) or SW504 (Δ*difA*, EPS^−^) cells were placed on polystyrene surfaces submerged in 1% methylcellulose medium and cell movements were recorded by time lapse photography. Continuous images were taken at 10 s intervals for 30 min. The motility and tracks of two poles of one representative isolated cell were analyzed. Data are presented as tracking plots (upper channels, trajectory for 30 min) and as velocity profiles (lower panel). Red and grey lines represent the results of tracking different poles of the same cell, respectively. Dark grey frames in the upper panels indicate the identified tethering events (see supplementary videos). The instantaneous velocity of cell pole (in lower panel, red and grey lines represent two poles of one cell, respectively) was calculated by measuring the distance from a starting to an ending point within two frames of TIFF image sequences.

In a liquid medium containing 1% methylcellulose, the TFP-driven S motility of *M. xanthus* dominates and A motility is not active^6^,^9^. For this reason, cells can be tracked in either A^+^ or A^−^ background with comparable results^19^. In order to investigate the effects of EPS production on single-cell S motility, isolated cells of wild-type strain DK1622 (EPS^+^), EPS deficient strain SW504 (EPS^−^, Δ*difA*) and EPS overproducing strain DK3088 (EPS^++^, *stk*)^20^,^21^ were used. Representative behavior of horizontally attached cells in each data set was manually selected and tracked in 1% methylcellulose. The trajectory and velocity profiles for one representative cell of each strain are shown in Fig. 1. During the observation period of 30 minutes, the selected cells exhibited different single-cell motility patterns. Horizontally oriented cells with less EPS moved with higher instantaneous velocity (lower panel of Fig. 1) and lower directional persistence (upper panel of Fig. 1). Moreover, more tethering events with cells in a vertical orientation were observed in the motion of SW504 cell (indicated with dark grey frames in upper panel of Fig. 1). Consistent with the previous observation made from 20 tracked cells, SW504 cells exhibited a rapid motility phenotype compared with that of DK1622 cells^9^. To statistically analyze the behaviors of horizontally and vertically oriented cell populations, community tracking algorithms^12^,^13^ were employed to further investigate large sample populations with single-cell resolution.

### EPS production strongly impacts the occurrence of vertically oriented, tethered *M. xanthus* cells in methylcellulose medium

**Figure 2:**
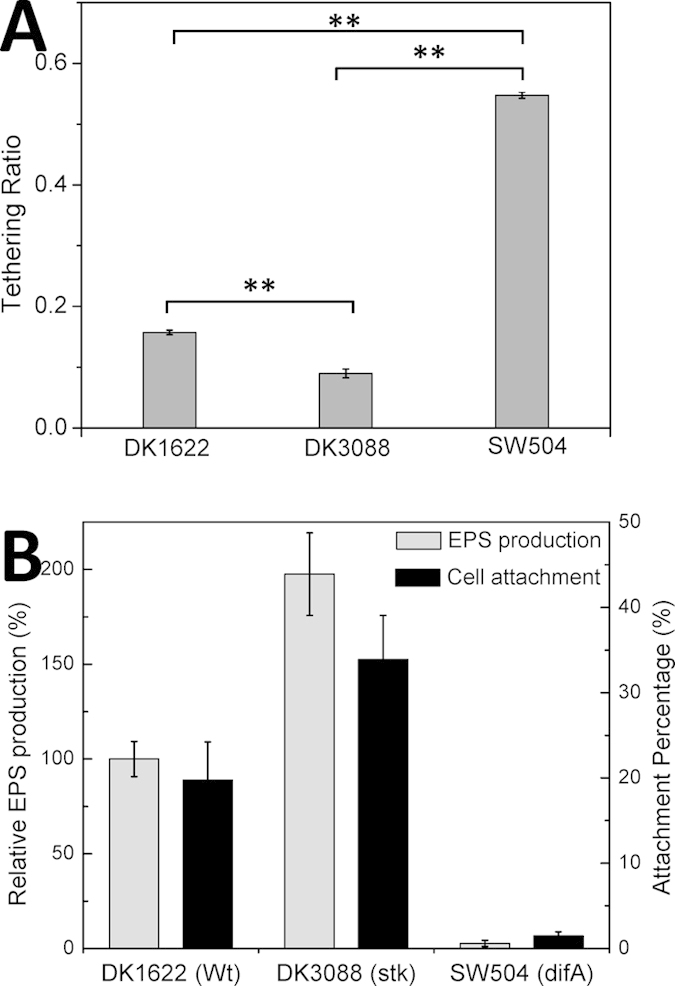
Tethering ratio and cell adhesiveness of *M. xanthus* cells. (**A**) The percentage of tethered vs. total cells of DK1622 (Wt), DK3088 (*stk*) or SW504 (Δ*difA*) were calculated on polystyrene surfaces in 1% methylcellulose. The means ± SD are plotted, Student’s *t*-test was employed for statistical analysis and **P < 0.01. (**B**) Quantitative analysis (grey columns) of EPS production of strain DK1622, DK3088 and SW504. Values for all strains were normalized to the wild-type DK1622. Attachment percentages (black columns) of DK1622, DK3088 and SW504 cells on a polystyrene surface in 1% methylcellulose were measured with the cell adhesion assay described in the *Materials and Methods*. The data represent triplicate experiments, and the means ± SD are plotted.

*M. xanthus* cells in methylcellulose medium exhibit tethering behavior, in which cells attach to a polystyrene surface by the tips of their pili and stand up from the surface^6^,^22^, but exhibit no lateral movement. The percentage of tethered cells was calculated over every frame in the acquisition, and approximately 900 frames were randomly chosen from the videos (see Methods) and analyzed for each strain (N = 38888 WT cell images, 7113 DK3088 cell images and 11160 SW504 cell images, respectively). As shown in Fig. 2A, the tethering ratio of SW504 (EPS^−^) cells was approximately 3 times higher than that of DK1622 (EPS^+^) cells, whereas DK3088 (EPS^++^) cells showed lower tethering ratio than DK1622 (EPS^+^) cells. Because EPS plays a key role in cell-substratum adhesion^23^, we measured the adhesiveness of *M. xanthus* cells on polystyrene surfaces in 1% methylcellulose together with their EPS production. As shown in Fig. 2B, cells producing more EPS exhibited stronger attachment on the polystyrene surfaces, which could be attributed to the additional adhesiveness provided by more EPS.

### The influence of EPS production on the velocity distribution of single-cell S motility of horizontal *M. xanthus*

**Figure 3:**
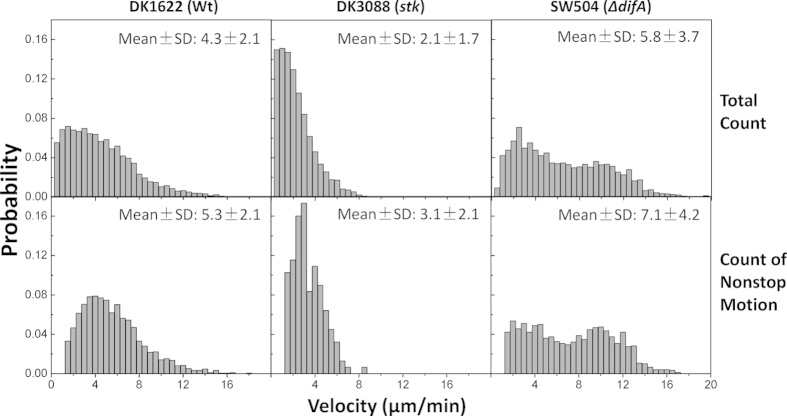
Velocity histograms of DK1622 (Wt), DK3088 (*stk*) and SW504 (Δ*difA*) cells on polystyrene in 1% methylcellulose. The upper panels show the histograms of the velocities of the full trajectories of all the motile cells; the lower panels show the trajectories when individual nonmotile (slower than 1 μm/min) segments are excluded.

It is known that EPS mediates *M. xanthus* cell attachment. Adhesion generally involves forces perpendicular rather than forces parallel to the surface, which are more relevant for surface motility. It is interesting to see how EPS impacts lateral motion along the polystyrene surface. We calculated the velocity distribution histograms of cells of three *M. xanthus* strains by tracking motile cells of each strain over 15 minutes (N = 4395 WT cells, 730 DK3088 cells and 829 SW504 cells, respectively). The observed distributions are complex, but a few comparisons can be made. If we compare the velocities at the peak of each distribution, EPS^−^ cells (SW504) showed slightly faster motion than the EPS^+^ WT (DK1622) cells, whereas EPS^++^ (DK3088) cells exhibited slightly slower motion than WT cells (upper panel, Fig. 3). These velocity differences, although clearly present, are surprisingly small for the three strains, especially given the large differences in surface adhesion noted in Fig. 2B. SW504 cells in particular displayed a broad velocity histogram, in contrast with the more sharply peaked WT and DK3088 histograms, which indicated a tendency to move at a peak velocity and are relatively close in magnitude. Moreover, the tethering percentages of the three strains were different (Fig. 2A). To better determine the motility behavior of horizontal cells (which are more relevant to S-motility), we calculated the velocity histograms after excluding the segments of the tracks around times where the bacteria stopped and moved slower than 1 μm/min, which effectively filtered out the vertically tethered cells, and the fraction of which are significantly different for the EPS^+^ (DK1622) and EPS^++^ (DK3088) cells. One can see that the velocity distribution for DK1622 is peaked at a higher nominal velocity than the corresponding parameter for DK3088 and that the velocity distribution of DK1622 has high velocity tails that are absent in that of DK3088 (Fig. 3). Meanwhile, the EPS^−^ Δ*difA* (SW504) strain has a very broad velocity histogram.

To assess whether the overexpression of EPS has a homogenizing effect on the velocity distribution of *M. xanthus*, we statistically tested the difference between DK1622 and DK3088 velocity distributions by performing an analysis of the Pearson moments of the velocity distributions and calculating their skewness and kurtosis, which are statistical measures of the asymmetry and the ‘peakedness’ of a given distribution respectively. As shown in Table 1, the skewness was positive for all distributions because they were all right-skewed and the velocities were all non-negative. The key difference between the DK1622 and the DK3088 distribution is the higher kurtosis value of the DK3088 distribution, which indicates that the velocity distribution for DK3088 is much more sharply peaked than that for WT (DK1622). To further assess whether underproduction of EPS indeed results in a high velocity subpopulation of *M. xanthus* cells, we statistically tested the difference between DK1622 and SW504 velocity distributions using the Larkin test, an algorithm for assessing bimodality vs. unimodality in a univariate distribution based on the F-test. In this context, a larger value of the bimodality index indicates greater evidence for rejecting the null hypothesis of unimodality. Because the Larkin test is based on the F-test, it allows for a statistical significance evaluation. The bimodality indexes are shown in Table 1, and we obtained a *p*-value of just over 0.01 for SW504 and just under 0.01 for moving SW504. This analysis indicates that the velocity distribution of SW504 is quantitatively different than that for DK1622, with the EPS underproducer having a significantly more bimodal distribution.

### *M. xanthus* EPS affects the directional persistence of single-cell S motility

**Figure 4:**
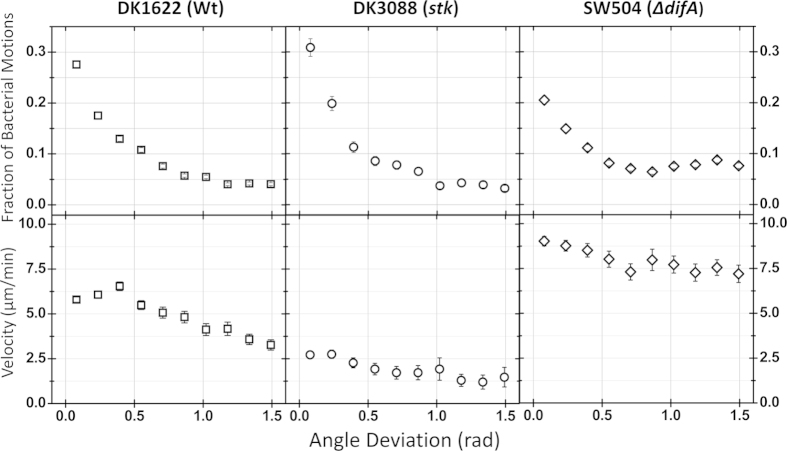
Angle deviation of DK1622 (Wt), DK3088 (*stk*) and SW504 (Δ*difA*) cells on polystyrene in 1% methylcellulose. The angle deviation was defined as the angle between the instantaneous velocity and the long axis of the cell body and was measured as described in the *Materials and Methods*. At each time point, when the cell is moving exactly along its body axis, the value of angle deviation is 0; when it moves perpendicular to its body axis, the value of angle deviation is π/2. The upper panels show the fraction of motions taken at different angle deviations, and the lower panels show the velocity of motion at different angle deviation.

**Figure 5:**
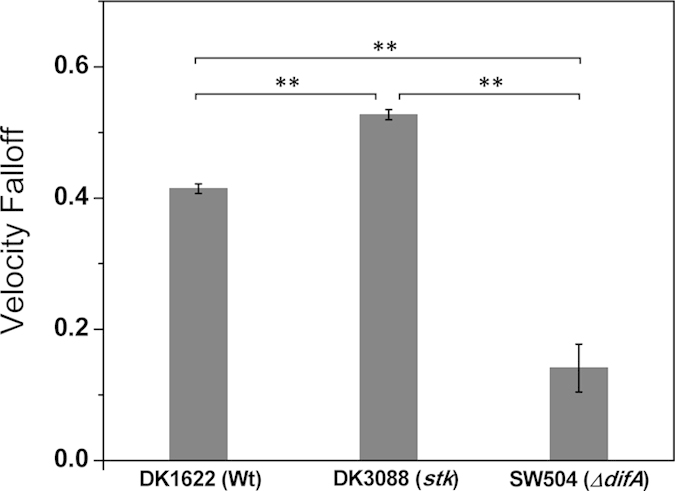
Velocity falloff of strain DK1622 (Wt), DK3088 (*stk*) and SW504 (Δ*difA*) on polystyrene in 1% methylcellulose. The velocity falloffs (mean ± SD) of different *M. xanthus* cells were calculated as described in the *Materials and Methods* and quantified the loss of cell velocity when moving perpendicular to the cell body axis. The WT and *stk* strains moved faster along their body axis, whereas the Δ*difA* strain showed very little change. Student’s *t*-test was employed for statistical analysis and **indicates P < 0.01.

EPS^−^ cells also showed low preference for moving along their body axis in single-cell motility, whereas EPS^+^ cells preferentially and consistently moved lengthwise along their respective body axes (Fig. 1). We quantified the extent of the sideways motion of cells (N = 4395 WT cells, 730 DK3088 cells and 829 SW504 cells, respectively) by measuring the parameter of angle deviation, defined as the angular difference between the long axis and the moving direction of a cell. Cells of all three strains were more likely to move along their body axis, at low angle deviation (Fig. 4, upper panels). This effect was less pronounced in the SW504 (EPS^−^) strain, which had fewer cells moving at the lowest angle deviation but larger fractions of cells moving at a high angle deviation. The frequency distributions of motions taken at different angle deviations in SW504 was significant different with that of DK1622 and DK3088 (both P < 0.05 in K-S test), whereas the difference between distributions of DK1622 and DK3088 was not large enough to reach significance at the P = 0.05 level in K-S test (0.05 < P < 0.10). Cells moved faster when moving along their body axis and at low angle deviation. Moreover, SW504 (EPS^−^) cells moved at nearly the same velocity while moving at a high angle deviation (perpendicular to their body axis), whereas WT (EPS^+^) and DK3088 (EPS^++^) cells moved faster when moving along their body axis and at a low angle deviation (Fig. 4, lower panels). We further quantified this phenomenon by calculating the ratio between their velocity at low angle deviation and at high angle deviation, which was defined as the velocity falloff (Fig. 5, N = 4395 WT cells, 730 DK3088 cells and 829 SW504 cells, respectively). DK1622 (EPS^+^) cells lost approximately 41% of their velocity when moving perpendicular to their body axis, and DK3088 (EPS^++^) cells were less able to do so. Conversely, the EPS^−^ cells (SW504) showed a very small velocity falloff (less than 18%), meaning that cells lost very little instantaneous velocity when moving sideways.

## Discussion

In this study, we investigate the single-cell TFP-driven motility that underlies collective, multi-cell S-motility of *M. xanthus*, without the complicating mutual influence of neighboring cells on a given cell’s individual motility. To do so, we measured several motility parameters exhibited by single cells of *M. xanthus* in methylcellulose medium by tracking every cell in a library of microscopy videos with community tracking algorithms^13^,^17^,^18^. The average velocity is normally considered an important parameter of kinematics or motility. The data show that the specific influence of EPS on TFP-driven motility is more subtle than previously thought, and impacts a range of motility parameters. The nature of this influence can be seen in the calculated velocity distribution histograms of *M. xanthus* cells. Interestingly, the velocity distributions for DK1622 and DK3088 both peaked at velocity values that are not too different from one another, with DK3088 having a slightly lower peak velocity.

Increased EPS secretion can result in both more surface coverage by EPS and/or thicker deposits of EPS, depend on the wetting properties of EPS on the surface, which depend on both surface and interface energies, as well as van der Waals forces. Simply having more EPS coverage on the surface but at the same thickness of EPS would not lead to a significant slow-down of single-cell motility. However, more EPS secretion in the form of thicker EPS can lead to increased viscous drag. In the present context, data are consistent with more EPS secretion in the form of a slightly thicker wetting layer, but at significantly greater EPS surface coverage. The macromolecular organization of EPS may also contribute to the lack of a simple correlation between surface adhesion and resistance to surface motility. The biggest influence of EPS appears to be the shapes and widths of the velocity distributions. The distribution for DK1622 is asymmetric, with a slowly decaying ‘tail’ from having more cells at higher velocities; these high velocity tails are absent in the distribution for DK3088, which is more tightly and symmetrically centered around the peak velocity value (Fig. 3). These observations were confirmed by the Pearson moments statistical analysis (Table 1), which suggests that EPS may play a role in coordinating and homogenizing multicellular motility. Interestingly, the narrowing of the distribution and decrease of the mean in the overexpressing DK3088 mutant implies that the drag force is velocity-dependent (impacting higher cell velocities more than it does lower cell velocities), consistent with fluid friction rather than solid friction on a surface, which in contrast, depends on the normal force and has no (or only weak) velocity dependence.

In the absence of EPS, which TFP are known to bind to, individual cells are able to move faster in the methylcellulose medium. Moreover, SW504 cells have a broad velocity histogram that appears to be bimodal, with a subpopulation of fast cells that are more than 2.5× faster than the slow population, in contrast with the behavior of WT and DK3088, both of which seemed to be peaked around a single, albeit different, velocity value. This observation is further supported by the analysis of histograms using the Larkin test (Table 1). Having no EPS causes the SW504 cell population to bifurcate into a high velocity subpopulation and a low velocity subpopulation, rather than to simply have a broader distribution about a higher mean value, which is the expectation if we had only a lower degree of velocity dependent drag from fluid friction in the EPS underproducer.

We hypothesize that not all TFP-EPS binding events lead to pilus retraction and force generation and that EPS has a homogenizing influence on the cell velocities. Consistent with this, SW504 cells generally move at disparate speeds, and may not be able to coordinate into groups of cells at homogeneous velocities. By comparing all three strains, the trend indicates that greater secretion of EPS progressively eliminates the high velocity subpopulation of motile cells rather than slowing all cells equally. There are a number of mutually compatible explanations for the existence of subpopulations with different average velocities. For the EPS-deficient mutant SW504, a significant subpopulation of cells may have TFP that can bind directly to the ‘naked’ solid surface directly rather than to the viscoelastic polymeric EPS, which results in more direct force transduction and thereby a faster population of cells. EPS secretion may also lead to intracellular signaling that inhibits high motility, and an optimum level of EPS secretion may homogenize velocity distributions in a manner that is amenable to S-motility.

To test the hypothesis that EPS can homogenize cell velocities, we investigated two additional cell motility parameters, the angle deviation and velocity falloff, which quantitatively characterize the directional persistence of *M. xanthus* cells in methylcellulose medium. All three strains were more likely to move along their body axis (Fig. 4, upper panels), but the SW504 (EPS^−^) strain exhibited a systematically greater tendency to have more cells move at higher angles of deviation away from the cell axis. In addition, it was observed that velocity falls off faster for WT cells moving at finite angle deviations in WT strains than for EPS^−^, which is less sensitive, as seen in the percentage change of the falloff (18% vs. 41%). Together, these observations are consistent with a picture where EPS-TFP binding keeps *M. xanthus* ‘on track’, with less movement away from the cell axis and a smaller velocity falloff. In *P. aeruginosa*, it has been shown that motile cells deposit a trail of Psl exopolysaccharide on solid surfaces by twitching motility, and the subsequent cells that encounter these trails pull themselves towards the Psl-rich regions by their TFP with higher probability^15^. Moreover, in the photosynthetic cyanobacterium *Synechocystis* sp. PCC 6803, which has the ability to perform light-directed motility over solid surfaces, cells at the front of a moving finger-like projection secrete and leave behind an extracellular substance trail that is subsequently followed by other cells, which suggests that the extracellular substance modifies the physical properties of the substrate and leads to enhanced motility by guiding the dynamic spatial organization of multicellular communities^24^.

The behavior of the vertically tethered *M. xanthus* cells was observed in the methylcellulose medium^6^, and constitutes direct evidence for bacterial pilus retraction in the context of motility^22^. We find that the SW504 (EPS^−^) strain has systematically higher fractions of cells in the vertically tethered orientation. Similar effects are observed for *P. aeruginosa*, where it is found that mutants with decreased levels of exopolysaccharide Psl secretion have a higher fraction of standing cells^13^. We hypothesized that *M. xanthus* EPS promotes horizontal adhesion at the expense of the vertical population and that direct interactions between TFP and the bare surface may enhance the probability that cells are vertical tethered. Consistent with this, there is a 5× difference in the standing fraction when SW504 (EPS^−^) and DK3088 (EPS^++^) are compared.

It is interesting to note that *M. xanthus* cells generally stopped moving in the horizontal plane when they were vertically tethered (Fig. 1), in contrast to the behavior of *P. aeruginosa*, where vertically oriented cells move diffusively on the surface and at high speed^12^,^13^. We hypothesize that this is due to the localization of active TFP and their associated directions of pull. Vertically tethered *M. xanthus* cells exhibit a simple up-down movement, which implies that the functioning TFP are pulling mostly in the vertical direction. This contrasts with vertical cells of *P. aeruginosa* which can ‘walk’ laterally via TFP and produces high speed diffusive movement^12^,^13^, which implies that the TFP can be splayed and pulled with a force component along the surface. Taken together, these results are highly suggestive. TFP in *M. xanthus* pull rigidly in directions that are generally parallel to the cell body, whereas TFP in *P. aeruginosa* can pull in a multiplicity of directions relative to the cell body. This observation is consistent with the fact that TFP in *M. xanthus* drive S motility, where cells follow one another with velocity vectors parallel to their cell bodies, whereas TFP in *P. aeruginosa* drive twitching motility, where cells move in a jittery random fashion with complex relations between velocity vectors and cell body axes.

The *M. xanthus* extracellular matrix (ECM) is mainly composed of carbohydrate substances (EPS) with associated proteins and nucleic acids^25^,^26^, and has important functions in motility, cell-cell cohesion and fruiting body formation^27^,^28^,^29^. It has been hypothesized that *M. xanthus* EPS may decrease the friction when S-motile cells move across a solid surface via the retraction of TFP^10^. The recent observation of aperiodic stick-slip movements in *M. xanthus* S motility due to TFP-generated force interacting with EPS indicates that EPS acts as much like a lubricant as it does like a glue^14^ when multicellular movements are considered. The lubricating function of *M. xanthus* EPS explains the observation that the highly motile cells that actively drive fruiting body formation move in the presence of high concentrations of EPS^14^,^28^,^30^.

The TFP-driven motility of *M. xanthus* is usually investigated on solid agar medium, in the form of S-motility. To elucidate the dynamic effects of EPS production on single-cell motility without the complexities of coordinated multicellular movement, we exploited the fact that A^−^S^+^ cells are able to move as isolated cells in the submerged liquid medium and tracked *M. xanthus* cells with large sample populations in the current study. Here, we demonstrated that EPS impacts the pattern of TFP-mediated single-cell motility in *M. xanthus*, influencing the velocity profile, tethering probability and directional persistence. Bacterial EPS normally forms viscous aqueous suspensions due to their large molecular mass and complicated interactions with other macromolecules, ions and cells, as well as with some low-molecular-mass solutes^31^. The rheological properties of isolated EPS from different *M. xanthus* cells suggest that EPS play fundamental roles in cellular agglutination and clumping in liquid cultures 32,33. On solid surface, EPS act as a physical substratum to maintain *M. xanthus* biofilm structure and functions^28^,^34^. The phenotypes of the mutants with alterations in different genetic loci responsible for *M. xanthus* EPS production examined in this study suggested that the surface attachment of cells significantly depends on the amount of EPS. Consistent with this observation, greater average adhesion force of rupture events than forces obtained on most other microbial surfaces has been determined in *M. xanthus* by *in vivo* force spectroscopy using atomic force microscopy (AFM), and longer retraction length has been observed on the EPS-overproducing mutant DK3088 (*stk*) cells than for wild-type cells^35^. Further investigations will be aimed at obtaining better insight into the mechanism responsible for the coordination of grouped cell S motility by EPS through the balance of its lubricant and adhesive functions.

## Materials and Methods

### Bacterial growth conditions and strains

The *M. xanthus* strains used in this study were wild-type strain DK1622^36^, EPS deficient strain SW504 (Δ*difA*)^20^ and EPS overproducing strain DK3088 (*stk*)^21^. *M. xanthus* were grown in CYE medium^37^ at 32 °C on a rotary shaker at 300 rpm for 24 hr and were harvested by 13,000 × g centrifugation for 5 min.

### Methylcellulose assay for motility

The TFP-mediated single-cell motility of *M. xanthus* was assayed as previously described^6^,^9^. The harvested *M. xanthus* cells were diluted in MOPS buffer (10 mM MOPS, 8 mM MgSO_4_, pH 7.6) to 5 × 10^6^ cell/ml, and 5 μl suspension was transferred into 12-well polystyrene plates (Costar, Fisher Scientific, USA) for 10 min. Then, the cells were overlaid with 500 μl of 1% methylcellulose in MOPS buffer, and placed at room temperature in the dark for 1 h before filming.

### Microscopic imaging

Cell movements were monitored with a Nikon Eclipse TE200 inverted microscope at ×60 or ×40, captured with a SPOT RT740 CCD camera (Diagnostic Instrument, USA) and recorded with SPOT advanced software (Version 4.6, Diagnostic Instrument). Recording was set at 10 s intervals and a TIFF image sequence file was outputted. The image scales were calculated by ImageJ software^38^ with a calibration scale. Microsoft videos were exported at 50 frame/s with SPOT software, which was 500 times faster than real-time.

### Bacteria tracking and motility analysis

In our previous work on *M. xanthus*, we used high speed tracking to investigate the spatiotemporal fine structure of motility exhibited by single cells or doublets of cells at short times^14^. Here, we use community tracking algorithms and population analysis over many hours, similar to those previously used for *P. aeruginosa*^12^,^13^. In total 13 videos of DK1622, 13 videos of DK3088 and 11 videos of SW504 were analyzed, for which the continuous images were taken at 10 s intervals for 30–60 min. We preprocessed the image by inverting it to make the bacteria brighter than the background and then applied a spatial filter to remove noise. Pixels that were local maxima in at least three directions, or were brighter than a predetermined threshold were designated as bacteria pixels^18^, which were then computationally joined to reconstruct locations of all bacteria. A single position for each bacterium was calculated by averaging *x* and *y* positions of its pixels, and the orientation of each bacterium was calculated from the moments of its backbone distribution^17^. To determine the length and width of the bacterium, we first rotated each bacterium by its orientation angle and then calculated the maximum *x* and *y* distances between any pair of pixels. Bacteria positions were joined into tracks with the use of code written in IDL (ITT VIS, White Plains, NY)^18^. As a control analysis, representative motility trajectories and velocity profiles were generated by Manual Tracking^39^ as previously described^9^.

The five motility parameters listed below were calculated and further analyzed to characterize the TFP-mediated single-cell motility of different *M. xanthus* strains in 1% methylcellulose medium.

1) Velocity: From the cell trajectories, the instantaneous velocities (*v*_n_) for each frame of each cell were calculated via *v*_n_ = |X_n + 1_-X_n_|/10, where X_n_ is the cell position in frame *n*.

2) Velocity histogram: To calculate a velocity histogram, we first separated out cells that moved with an average velocity of over 1.0 μm/min, which ensured that we measured the velocity of an actively moving cell rather than noise due to low amplitude motions of surface-attached cells about their equilibrium positions with average velocities near zero^40^. The remaining velocities were binned in half-micron increments and plotted as a histogram for each strain. The significant difference between DK1622 and DK3088 velocity distributions was analyzed by the Pearson moments of the velocity distributions and by calculating the skewness and kurtosis of the distributions. The significant difference between the DK1622 and SW504 velocity distributions was analyzed using the Larkin test^41^.

3) Tethering ratio: To detect bacteria that were tethered to the surface by one end, we used a cutoff based on the length of *M. xanthus* cells. Tethered cells would occasionally orient themselves normal to the surface (in a vertical orientation), thus having an apparent length that was significantly shorter than that of a horizontally oriented motile cell. At the same time, tethered cells would move less than their body length over the course of the acquisition. The tethering ratio was then calculated by the percentage of tethered cells versus total cells as previously described^9^,^42^.

4) Angle deviation: We defined the angle deviation of a *M. xanthus* cell to be the angle between the instantaneous velocity and the long axis of the cell body. From the position of a cell in a frame *n*, the direction of the long axis of this cell was determined by the straight line between the two ends of the cell. The direction of motion was determined by the straight line between the leading end of the cell in frame *n* and the same leading end in frame *n* + *1* (10 s interval). At each time point when the cell is moving exactly along its body axis, a value of 0 is assigned; when it moves perpendicular to its body axis, a value of π/2 is assigned. The significant difference between the distributions of motions taken at different angle deviations was analyzed by the Kolmogorov-Smirnov (K-S) test.

5) Velocity falloff: As *M. xanthus* cells prefer to move in the direction of their body axis^2^, it is expected that they would move faster when moving at low angles of deviation between the velocity vector and the body axis. For each strain, we can measure the average velocity (*v*_A_) for the time points when the cell is moving along its body axis (to within π/6) and the average velocity (*v*_P_) for the time points when the bacterium is moving perpendicular to its body axis (to within π/6). The velocity falloff (*VF*) was calculated via *VF* = (*v*_A_–*v*_P_)/*v*_A_.

### EPS quantification

EPS production of different strains in CYE liquid was measured using a trypan blue binding assay^43^. The relative amounts of EPS were calculated relative to trypan blue bound to the wild-type DK1622 cells. Triplicate experiments were performed.

### Cell adhesion assay

This assay was adapted from the previously published procedure^44^. First, 10 μl *M. xanthus* cells at 1 × 10^8^ cell/ml in MOPS buffer was added into 96-well polystyrene plates (Costar, Fisher Scientific) for 10 min, and overlaid cells with 100 μl of 1% methylcellulose in MOPs buffer. The cells were placed at room temperature in the dark for 1 h. Then, 100 μl 5% glutaraldehyde was added and methylcellulose solution were gently removed. The nonadherent and loosely attached cells were gently washed with two 100 μl aliquots of MOPS buffer. The attached cells were fixed by the addition of 100 μl 5% glutaraldehyde for 20 min at room temperature. Samples were washed with 500 μl MOPS buffer 3 times, stained with 100 μl 0.1% crystal violet for 60 min, and solubilized in 100 μl 10% acetic acid, and the absorbance was measured at 570 nm. Next, 50 μl MOPS buffer was used as blank, and 10 μl cells at 1 × 10^8^ cell/ml was directly stained with 100 μl 0.1% crystal violet for 60 min. The cell pellet collected by 5,000 × g centrifugation for 10 min was dissolved in 100 μl 10% acetic acid, and the absorbance at 570 nm was used to estimate total biomass. Triplicate experiments were performed.

## Additional Information

**How to cite this article**: Hu, W. *et al.* Interplay between type IV pili activity and exopolysaccharides secretion controls motility patterns in single cells of *Myxococcus xanthus. Sci. Rep.* **6**, 17790; doi: 10.1038/srep17790 (2016).

## Supplementary Material

Supplementary Information

Supplementary Video S1

Supplementary Video S2

Supplementary Video S3

Supplementary Video S4

Supplementary Video S5

Supplementary Video S6

## Figures and Tables

**Table 1:** Statistical analysis of velocity histograms shown in Fig. 3.

**Pearson Moments Analysis**				**Larkin Test**		
**Strain**	**Count**	**Skewness**	ExcessKurtosis	**Strain**	**Count**	BimodalityIndex
DK1622(WT)	Total	0.42	−0.47	DK1622(WT)	Total	1.3
	Nonstop	0.44	−0.47		Nonstop	1.2
DK3088(*stk*)	Total	3.4	16	SW504(Δ*difA*)	Total	2.1
	Nonstop	3.6	15		Nonstop	2.2

## References

[b1] Shi W. & Lux R. Focal adhesion: getting a grasp on myxobacterial gliding. Nat Chem Biol 3, 205–206 (2007). 17372604 10.1038/nchembio0407-205

[b2] Hartzell P., Shi W. & Youderian P. Gliding motility of Myxococcus xanthus (ed. Whitworth D. E.) Ch. 6 103–122 (American Society for Microbiology Press, 2008).

[b3] Hodgkin J. & Kaiser D. Genetics of gliding motility in *Myxococcus xanthus*: two gene systems control movement. Mol. Gen. Genet. 171, 177–191 (1979).

[b4] Hodgkin J. & Kaiser D. Genetics of gliding motility in *Myxococcus xanthus*: genes controlling movement of single cells. Mol. Gen. Genet. 171, 167–176 (1979).

[b5] Wu S. S., Wu J. & Kaiser D. The *Myxococcus xanthus* pilT locus is required for social gliding motility although pili are still produced. Mol Microbiol 23, 109–121 (1997). 9004225 10.1046/j.1365-2958.1997.1791550.x

[b6] Sun H., Zusman D. R. & Shi W. Type IV pilus of *Myxococcus xanthus* is a motility apparatus controlled by the frz chemosensory system. Curr Biol 10, 1143–1146 (2000). 10996798 10.1016/s0960-9822(00)00705-3

[b7] Mattick J. S. Type IV pili and twitching motility. Annu Rev Microbiol 56, 289–314 (2002). 12142488 10.1146/annurev.micro.56.012302.160938

[b8] Li Y. *et al.* Extracellular polysaccharides mediate pilus retraction during social motility of *Myxococcus xanthus*. Proc Natl Acad Sci USA 100, 5443–5448 (2003). 12704238 10.1073/pnas.0836639100 PMC154364

[b9] Hu W. *et al.* Exopolysaccharide-independent social motility of *Myxococcus xanthus*. PLoS One 6, e16102 (2011). 21245931 10.1371/journal.pone.0016102 PMC3016331

[b10] Lancero H. *et al.* Characterization of a *Myxococcus xanthus* mutant that is defective for adventurous motility and social motility. Microbiology 150, 4085–4093 (2004). 15583161 10.1099/mic.0.27381-0

[b11] Lu A. *et al.* Exopolysaccharide biosynthesis genes required for social motility in *Myxococcus xanthus*. Mol Microbiol 55, 206–220 (2005). 15612929 10.1111/j.1365-2958.2004.04369.x

[b12] Gibiansky M. L. *et al.* Bacteria use type IV pili to walk upright and detach from surfaces. Science 330, 197 (2010). 20929769 10.1126/science.1194238

[b13] Conrad J. C. *et al.* Flagella and pili-mediated near-surface single-cell motility mechanisms in *P. aeruginosa*. Biophys J 100, 1608–1616 (2011). 21463573 10.1016/j.bpj.2011.02.020 PMC3072661

[b14] Gibiansky M. L., Hu W., Dahmen K. A., Shi W. & Wong G. C. Earthquake-like dynamics in *Myxococcus xanthus* social motility. Proc Natl Acad Sci USA 110, 2330–2335 (2013). 23341622 10.1073/pnas.1215089110 PMC3568354

[b15] Zhao K. *et al.* Psl trails guide exploration and microcolony formation in *Pseudomonas aeruginosa* biofilms. Nature 497, 388–391 (2013). 23657259 10.1038/nature12155 PMC4109411

[b16] Xie J., Khan S. & Shah M. Automatic tracking of *Escherichia coli* bacteria. Med Image Comput Comput Assist Interv 11, 824–832 (2008). 18979822 10.1007/978-3-540-85988-8_98

[b17] Mohraz A. & Solomon M. J. Direct visualization of colloidal rod assembly by confocal microscopy. Langmuir 21, 5298–5306 (2005). 15924453 10.1021/la046908a

[b18] Crocker J. C. & Grier D. G. Methods of digital video microscopy for colloidal studies. J Colloid Interf Sci 179, 298–310 (1996).

[b19] Higgs P. & Merlie J. J. Myxococcus xanthus: cultivation, motility, and development (ed. Whitworth D. E.) Ch. 27 465–478 (American Society for Microbiology Press, 2008).

[b20] Yang Z., Geng Y., Xu D., Kaplan H. B. & Shi W. A new set of chemotaxis homologues is essential for *Myxococcus xanthus* social motility. Mol Microbiol 30, 1123–1130 (1998). 9988486 10.1046/j.1365-2958.1998.01160.x

[b21] Dana J. R. & Shimkets L. J. Regulation of cohesion-dependent cell interactions in *Myxococcus xanthus*. Journal of bacteriology 175, 3636–3647 (1993). 8501067 10.1128/jb.175.11.3636-3647.1993 PMC204765

[b22] Kaiser D. Bacterial motility: how do pili pull? Curr Biol 10, R777–780 (2000). 11084348 10.1016/s0960-9822(00)00764-8

[b23] Arnold J. W. & Shimkets L. J. Cell surface properties correlated with cohesion in *Myxococcus xanthus*. J. Bacteriol. 170, 5771–5777 (1988). 3142857 10.1128/jb.170.12.5771-5777.1988 PMC211681

[b24] Ursell T., Chau R. M., Wisen S., Bhaya D. & Huang K. C. Motility enhancement through surface modification is sufficient for cyanobacterial community organization during phototaxis. PLoS computational biology 9, e1003205 (2013). 24039562 10.1371/journal.pcbi.1003205 PMC3763999

[b25] Behmlander R. M. & Dworkin M. Biochemical and structural analyses of the extracellular matrix fibrils of *Myxococcus xanthus*. J Bacteriol 176, 6295–6303 (1994). 7929001 10.1128/jb.176.20.6295-6303.1994 PMC196971

[b26] Hu W. *et al.* DNA builds and strengthens the extracellular matrix in *Myxococcus xanthus* biofilms by interacting with exopolysaccharides. PLoS One 7, e51905 (2012). 23300576 10.1371/journal.pone.0051905 PMC3530553

[b27] Shimkets L. J. Correlation of energy-dependent cell cohesion with social motility in *Myxococcus xanthus*. J Bacteriol 166, 837–841 (1986). 2940231 10.1128/jb.166.3.837-841.1986 PMC215202

[b28] Lux R., Li Y., Lu A. & Shi W. Detailed three-dimensional analysis of structural features of *Myxococcus xanthus* fruiting bodies using confocal laser scanning microscopy. Biofilms 1, 293–303 (2004).

[b29] Konovalova A., Petters T. & Sogaard-Andersen L. Extracellular biology of *Myxococcus xanthus*. FEMS Microbiol Rev. 34, 89–106 (2010). 19895646 10.1111/j.1574-6976.2009.00194.x

[b30] Hendrata M., Yang Z., Lux R. & Shi W. Experimentally guided computational model discovers important elements for social behavior in myxobacteria. PLoS One 6, e22169 (2011). 21811570 10.1371/journal.pone.0022169 PMC3139613

[b31] Sutherland I. Biofilm exopolysaccharides: a strong and sticky framework. Microbiology 147, 3–9 (2001). 11160795 10.1099/00221287-147-1-3

[b32] Kim S. H., Ramaswamy S. & Downard J. Regulated exopolysaccharide production in *Myxococcus xanthus*. J Bacteriol 181, 1496–1507 (1999). 10049381 10.1128/jb.181.5.1496-1507.1999 PMC93539

[b33] Hu W. *et al.* Effects of exopolysaccharide production on liquid vegetative growth, stress survival, and stationary phase recovery in *Myxococcus xanthus*. J Microbiol 50, 241–248 (2012). 22538652 10.1007/s12275-012-1349-5 PMC3819231

[b34] Merroun M. L., Ben Chekroun K., Arias J. M. & Gonzalez-Munoz M. T. Lanthanum fixation by *Myxococcus xanthus*: cellular location and extracellular polysaccharide observation. Chemosphere 52, 113–120 (2003). 12729693 10.1016/S0045-6535(03)00220-0

[b35] Pelling A. E., Li Y., Shi W. & Gimzewski J. K. Nanoscale visualization and characterization of *Myxococcus xanthus* cells with atomic force microscopy. Proc Natl Acad Sci USA 102, 6484–6489 (2005). 15840722 10.1073/pnas.0501207102 PMC1088375

[b36] Kaiser D. Social gliding is correlated with the presence of pili in *Myxococcus xanthus*. Proc Natl Acad Sci USA 76, 5952–5956 (1979). 42906 10.1073/pnas.76.11.5952 PMC411771

[b37] Campos J. M., Geisselsoder J. & Zusman D. R. Isolation of bacteriophage MX4, a generalized transducing phage for *Myxococcus xanthus*. J Mol Biol 119, 167–178 (1978). 416222 10.1016/0022-2836(78)90431-x

[b38] Rasband W. S. ImageJ. National Institutes of Health, Bethesda, Maryland, USA (1997-2015). Available at: http://rsbinfonihgov/ij/.

[b39] Cordelières F. Manual Tracking, a plug-in for ImageJ software, Institut Curie, Orsay, France (2005).

[b40] Spormann A. M. & Kaiser A. D. Gliding movements in *Myxococcus xanthu*s. J Bacteriol 177, 5846–5852 (1995). 7592333 10.1128/jb.177.20.5846-5852.1995 PMC177408

[b41] Larkin R. P. An algorithm for assessing bimodality vs. unimodality in a univariate distribution. Behav Res Methods 11(4), 467–468 (1979).

[b42] Li Y., Lux R., Pelling A. E., Gimzewski J. K. & Shi W. Analysis of type IV pilus and its associated motility in *Myxococcus xanthus* using an antibody reactive with native pilin and pili. Microbiology 151, 353–360 (2005). 15699186 10.1099/mic.0.27614-0

[b43] Black W. P. & Yang Z. *Myxococcus xanthus* chemotaxis homologs DifD and DifG negatively regulate fibril polysaccharide production. Journal of bacteriology 186, 1001–1008 (2004). 14761994 10.1128/JB.186.4.1001-1008.2004 PMC344214

[b44] Humphries M. J. In Extracellular matrix protocols Vol. 139 Methods in Molecular Biology (eds Streuli Charles H. & Grant Michael E.) 279–300 (Humana Press, 2000).

