# Supplementary material for: Interplay between type IV pili activity and exopolysaccharides secretion controls motility patterns in single cells of Myxococcus xanthus
Source: Sci Rep. 2016 Jan 29;6:17790. doi: 10.1038/srep17790 (PMC4731782; doi:10.1038/srep17790)
Supplement: Supplementary Information [file srep17790-s1.pdf]

Interplay between type IV pili activity and exopolysaccharides secretion controls motility patterns in single cells of *Myxococcus xanthus*

Wei Hu, Maxsim L. Gibiansky, Jing Wang, Chuandong Wang, Renate Lux, Yuezhong Li, Gerard C. L. Wong, Wenyuan Shi

Supplementary information:

Video S1 *M. xanthus* DK1622 (Wt) cells were placed on polystyrene surface in MOPS medium containing 1% methylcellulose. The cell movements were recorded for 30 min and played 500X faster than real-time, and two poles of one representative isolated cell were respectively tracked with green and blue dots as described in *Materials and Methods*.

Video S2 Motility tracks of the selected cell in Video S1. Green and blue lines represent the results of tracking different poles of the same cell, respectively.

Video S3 *M. xanthus* DK3088 (*stk*) cells were placed on polystyrene surface in MOPS medium containing 1% methylcellulose. The cell movements were recorded for 30 min and played 500X faster than real-time, and two poles of one representative isolated cell were respectively tracked with green and blue dots as described in *Materials and Methods*.

Video S4 Motility tracks of the selected cell in Video S3. Green and blue lines represent the results of tracking different poles of the same cell, respectively.

Video S5 *M. xanthus* SW504 (*ΔdifA*) cells were placed on polystyrene surface in MOPS medium containing 1% methylcellulose. The cell movements were recorded for 30 min and played 500X faster than real-time, and two poles of one representative isolated cell were respectively tracked with green and blue dots as described in *Materials and Methods*.

Video S6 Motility tracks of the selected cell in Video S5. Green and blue lines represent the results of tracking different poles of the same cell, respectively.
